# Microbiota and Gut Inflammatory Markers (Zonulin and Fecal Calprotectin) Exhibit Age-Dependent Variation in Patients with Ulcerative Colitis

**DOI:** 10.3390/life15091412

**Published:** 2025-09-08

**Authors:** José Joaquín Merino, Nuría Bastande Rey, Rubén Fernández-García

**Affiliations:** 1 Instituto Pluridisciplinar (UCM) and Facultad de Farmacia, Departamento de Farmacología, Farmacognosia y Botánica, Universidad Complutense de Madrid (UCM), Paseo Ramon y Cajal s/n, 28040 Madrid, Spain; 2 Regenerative Medicine Group, Research Institute Hospital 12 de Octubre (imas12), 28041 Madrid, Spain; 3 Laboratorio Eurofins Megalab, 02003 Albacete, Spain; nbastande@sescam.jccm.es; 4 Department of Nursing, Physiotherapy and Medicine, University of Almeria, 04120 Almeria, Spain; rubenfer@ual.es

**Keywords:** vitamin D, bowel disease, inflammation, gut microbiota, enterocytes

## Abstract

Patients with bowel diseases (inflammatory bowel disease (IBD) in general) tend to seek medical, nursing, and/or physiotherapeutic consultations. Physiotherapists specialized in gastrointestinal (visceral) therapy can help reduce inflammation in patients with ulcerative colitis (UC). In this study, we divided UC patients into three groups according to their age: the youngest (18–35 years old), middle-aged (36–49 years old), and oldest (50–70 years old). Our hypothesis was that gut inflammatory markers (zonulin and fecal calprotectin levels) and microbiota strains would exhibit age-dependent variations in UC patients. We compared differences in zonulin, calprotectin, and vitamin D levels, together with a plethora of microbiota strains, based on age. Calprotectin is a marker of intestinal inflammation and zonulin identifies gut permeability; as IBD is characterized by gastrointestinal inflammation, these are useful markers for diagnosing and monitoring treatment/s in IBD patients, including ulcerative colitis (UC). Dysbiosis can alter the normal balance of intestinal function, and thus, several microbiota strains were compared between different age ranges in UC patients. The results indicated that the middle-aged UC (36–49) patients had the highest endogenous vitamin D levels, as well as lower zonulin and calprotectin levels than the youngest (18–35) and oldest (50–70) UC participants, respectively. The middle-aged group also had lower *Enterococcus*, *E. Coli biovare*, and *Pseudomonas* spp. levels than the youngest UC participants. Meanwhile, the most LPS microbiota producers were found in middle-aged patients. Finally, a higher number of Candida albicans and elevated LPS were found in the oldest UC participants than in the middle-aged (36–49) group. This study was, however, limited by uneven age-group sizes, which may have may limited the power in the youngest cohort. Although altered gut microbiota levels can increase gut inflammation in rodent models of UC, a definitive cause–effect relationship between UC and intestinal microbiota alteration is difficult to demonstrate in humans.

## 1. Introduction

Inflammatory bowel disease (IBD) is a disorder that provokes inflammation in the gastrointestinal tract over time (chronic process), mainly in the large/rectal intestine, especially in Crohn’s disease (CD) [1]. IBD encompasses two major conditions: ulcerative colitis (UC) with inflammation and CD. In both cases, patients report signs and symptoms including diarrhea, rectal bleeding, abdominal pain, weight loss, and fatigue [2]. From a pathophysiological perspective, genetic and environmental factors contribute to IBD pathogenesis (CD and UC) [3,4,5]. Additionally, disbalances in the number of intestinal microbiota (IM) can contribute to IBD pathology [6,7]. Diet is another important factor that affects the intestinal microenvironment and may regulate the function of gut microbiota. Vitamin D is a fat-soluble molecule that exerts immunomodulatory effects in IBD [8,9,10,11,12] and maintains the integrity of the intestinal barrier [13]. It is known that high serum levels of vitamin D (25 (OH) vitamin D) decrease the incidence of relapses of chronic intestinal inflammation [14,15,16] and can improve the clinical course of IBD [14]. Optimal vitamin D levels are between 50 to 75 nmol/L (20–30 ng/mL), although this varies depending on ethnicity and geographic location [17].

Several biomarkers in the blood for IBD diagnosis, such as Reactive Protein C (CRP), are useful at the clinical level. However, CRP levels vary in Crohn’s disease (CD) and decrease in UC patients [18]. The fecal calprotectin biomarker is a calcium and zinc binding protein that predicts clinical recurrence in patients with IBD [3,19,20,21]. Zonulin is considered a marker of intestinal permeability that regulates the tight junctions between cells in the walls of the gastrointestinal tract [22]. These markers are good indicators of intestinal inflammation and regulate innate immunity responses in autoimmune diseases [23,24]. On the other hand, the microbiota–gut–brain axis regulates the integrity of the intestinal epithelial barrier and affects intestinal permeability. Imbalances in the numbers of beneficial vs detrimental microbiota strains may lead to the accumulation of toxins in the intestine [25] which can then spread to other organs. For this reason, the composition and diversity of microbioma were analyzed in feces of UC patients in our study.

Our hypothesis is that gut inflammatory markers (zonulin and fecal calprotectin) and microbiota-strains will exhibit age-dependent variation in UC patients.

## 2. Aim

The aim of this research was to determine whether lower intestinal inflammatory markers (zonulin and calprotectin) would be found in middle-aged (36–49) UC patients with high endogenous vitamin D levels compared to young (15–35) or old (50–70) UC participants.

## 3. Material and Methods

### 3.1. Patient Study Groups

Patients with ulcerative colitis (UC) were divided into three groups according to their age. The youngest patients were 18–35 years old, with a mean ± S.E.M age of 32 ± 0.76; middle-aged UC patients were 36 and 49 (inclusive) years old, with a mean age of 43 ± 0.50; and the oldest UC participants were 50 and 70 (inclusive) years old, with a mean age of 69 ± 1.54.

### 3.2. Inclusion Criteria

Patients were selected according to the degree of their ulcerative colitis by an expert gastroenterologist. Chronic diarrhea was identified with fresh blood in almost 90% of patients, as well as severe bleeding from the lower part of the gastrointestinal tract, sudden and intense pushing of stool, expulsion of mucopurulent contents, and/or severe abdominal pain of a spasmodic nature, located mostly on the left side of the iliac fossa and that usually subsided after defecation. In patients with the proctitis form, symptoms were usually limited to the violent pushing of stool with the presence of fresh blood. The evolution of ulcerative colitis was also considered in our study.

### 3.3. Exclusion Criteria

Patients were excluded if they had obesity, metabolic disorders (e.g., thyroid disease, diabetes, Cushing syndrome), or autoimmune disorders. None of the subjects had received immunosuppressive drugs, nutritional supplementation with antioxidants, or vaccinations within at least 6 months prior to their inclusion in this study. Additionally, subjects with blood diseases, digestive problems, autoimmune diseases, hypertension, dermatological problems, or kidney/urological diseases were not considered for selection. Finally, women experiencing menopause, taking corticoids, receiving immunomodulatory drugs, or suffering from COVID-19 were also excluded.

### 3.4. Study Groups

Initially, 140 subjects were assessed for a possible diagnosis of ulcerative colitis (UC). Ultimately, 59 participants were excluded and 81 patients with a confirmed UC diagnosis were included. Included patients were then randomized as subjects in our study. This observational study included UC patients divided into three study groups according to age:

18–35 group, n = **15**: youngest patients (18–35 years old); included 9 women.

36–49 group, n = **38**: middle-aged patients (36–49 years old); included 23 women.

50–70 group, n = **28**: oldest patients (50–70 years old); included 12 women.

This observational study followed the Declaration of Helsinki (1974 and updated in 2000) and was approved by the Human Ethical Committee of the University of Almeria (code EFM 420.25). All participants signed the informed consent form; they did not receive financial compensation for their participation, and their anonymity was guaranteed throughout the study. Subjects were instructed about the procedures for providing samples and for microbiota analyses (feces). Participants were selected from a clinical database of patients, and efforts to minimize the number of patients were made.

**Figure 1:**
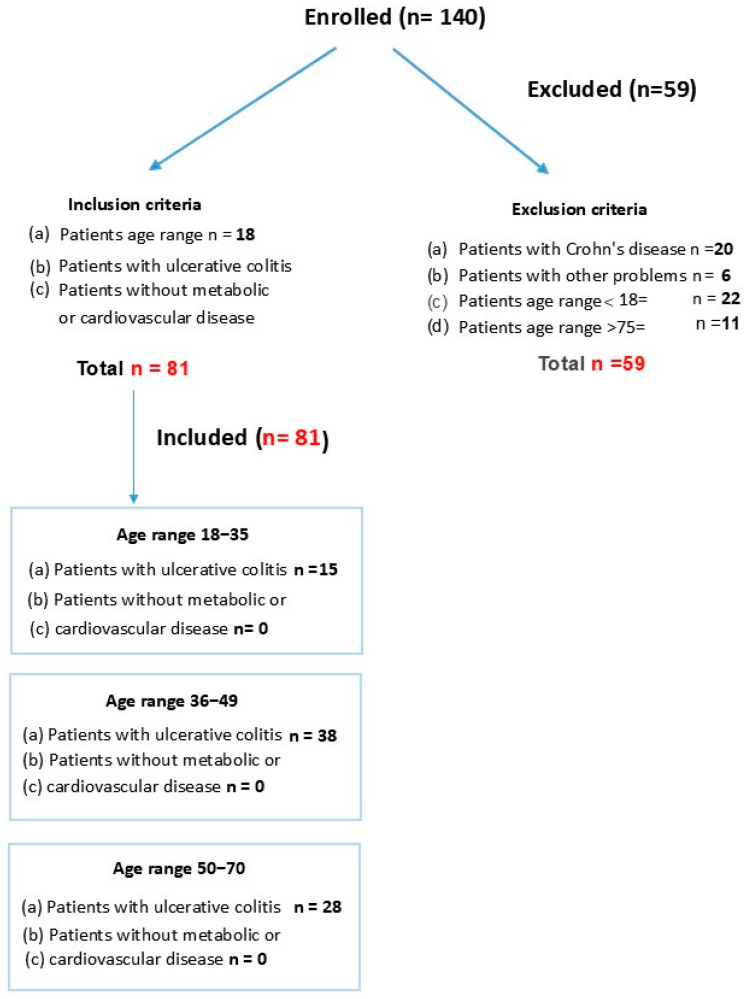
Age of patients with ulcerative colitis (UC).

The design for study groups is shown in Figure 1.

### 3.5. Measurement of PCR

Blood samples were collected in commercially available serum-separating tubes (vacutainer, Becton Dickinson, Europe). After collection, the blood was allowed to clot at room temperature for 30 min. Clots were then removed by centrifugation for 10 min at 200× *g* using a refrigerated centrifuge. Serum samples were stored at −80 °C immediately after their preparation [26].

### 3.6. Measurement of Calprotectin

Fecal samples were stored at −80 °C until shipment to the laboratory, where calprotectin analysis was performed. The level of calprotectin in the stool specimens was measured using a PhiCal Calprotectin ELISA kit (Immundiagnosik AG, Bensheim, Germany). The quantitative range was 0.65 to 84,000 ug/g after the appropriate dilution of fecal samples (to 1:50–1:1:100,000). The cutoff value in this study was set at 180 ug/g based on the results of the receiver operating characteristic curve analysis [27].

### 3.7. Measurement of Zonulin

Fecal zonulin concentrations were quantified by competitive ELISA assays. First, a biotinnylated zonulin tracer was added to all samples, including standards and calibrators. Aliquots of samples were then transferred and incubated in microtiter plate wells coated with polyclonal anti-zonulin antibodies. During incubation, the free target antigen competed with the biotinylated zonulin tracer for the binding of the polyclonal immobilized anti-zonulin antibodies on the microtiter plate wells. The unbound components were removed by washing with buffer three times. In a second incubation step, a streptavidin-labeled-peroxidase antibody was added to the wells, which bound to the biotinylated zonulin tracer. After the removal of unbound components, the peroxidase and tetramethylbenzidine (TMB) substrate were added to each well. Lastly, the enzymatic reaction was stopped by adding acidic stop solution. According to the manufacturer, a median concentration of 61 ng/mL (±46 ng/mL) fecal zonulin was estimated as “normal” for healthy subjects [26].

### 3.8. Measurement of Microbiota

Participants were asked to provide stool samples using an EasySampler^®^ Stool Collection Kit (ALPCO, Salem, NH, USA). Stool samples were homogenized using the Roche MagNA Lyser (Roche Inc., Basel, Switzerland). DNA was extracted from the homogenized fecal isolates using the QiaAmp PowerFecal DNA kit (Hilden, Germany). Bacterial profiles were determined by broad-range amplification and sequence analysis of 16S rRNA genes. Amplicons were generated using primers that target approximately 400 base pairs of the V3V4 variable region of the 16S rRNA gene. Pooled amplicons were quantified using a Qubit Fluorometer 2.0 (Invitrogen, Carlsbad, CA, USA). The pool was diluted to 4 nM and denatured with 0.2 N NaOH at room temperature. The denatured DNA was diluted to 15 pM and spiked with 25% of the Illumina PhiX control DNA prior to loading the sequencer. Illumina paired-end sequencing was performed on the MiSeq platform with versions v2.4 of the Miseq Control Software and of MiSeq Reporter using a 600 cycle version 3 reagent kitv. In all PCR reactions, which were carried out in a 30 μL reaction volume, the forward primer (341F: 5′-CCTACGGGNGGCWGCAG-3′) and the reverse primer (805R: 5′-GACTACHVGGGTATCTAATCC-3′) were utilized. Intestinal flora were classified at five taxonomic levels: genus, family, order, class, and phylum. Instrumental variables (IVs) were derived from SNPs meeting genome-wide suggestive significance (*p* < 1 × 10^−5^). To balance IV strength and independence, LD parameters were set at r^2^ < 0.01 with a 10 Mb clumping window. This stringent r^2^ threshold minimized LD bias while preserving statistical power. Finally, SNPs with ambiguous or palindromic alleles were excluded [27].

In detail, low-quality sequences were eliminated if their average quality score was less than the 20 bp threshold, while all high-quality sequence read longer than 250 bp were integrated. Subsequently, the sequences were clustered using USEARCH software (v10.0.240) and divided into non-singleton amplicon sequence variants (ASVs) using the non-clustered denoising method. Following this, taxonomic annotation of bacterial and archaeal 16S rRNA genes was performed using the SILVA 123 database. The study used the α diversity metrics (Chao1, Shannon, Ace) and β diversity metrics (PcoA, NMDS, ANOSIM).

Vsearch (v2.15.2) was used, and taxonomic annotation of bacterial and archaeal 16S rRNA genes was performed using the SILVA 123 database.

By employing the castor R package’s (4.1.3) hidden state prediction algorithm, gene family copy numbers were inferred by identifying sequences within this phylogenetic framework. Gene family copy numbers per sample were calculated by integrating sequence abundance profiles. These gene families were mapped to the Kyoto Encyclopedia of Genes and Genomes (KEGG) database to quantify functional abundances. The rfPermute R package (v2.5.4) was implemented to construct a random forest classification model utilizing differential abundant phylum and genus between groups. Key parameters were set as ntree = 500 and nrep = 1000 [27].

### 3.9. Measurement of Vitamin D

Blood samples were collected into commercially available serum-separating tubes (vacutainer, Becton Dickinson, Europe). Serum 25 (OH) D was measured by liquid chromatography/tandem mass spectrometry, which is the standard for the measurement of vitamin D and its components [28,29].

## 4. Results

Below is a summary of the observed Inflammatory mediators and number of LPS microbiota producers.

### 4.1. Zonulin

**Figure 2:**
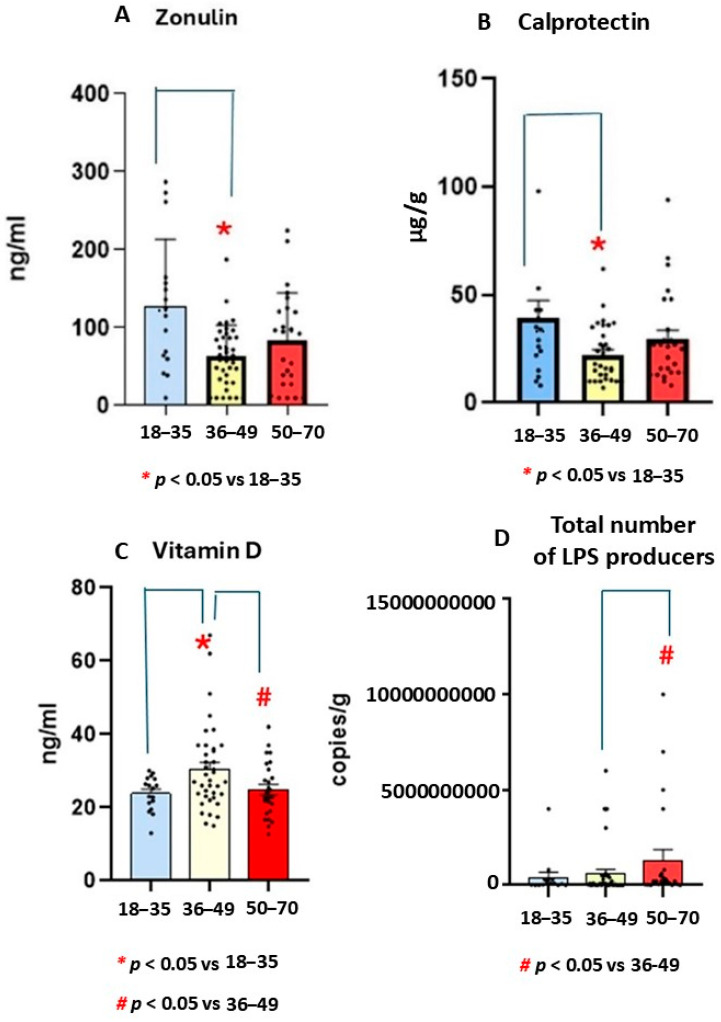
Mean values ± S.E.M for zonulin (ng/mL, (**A**), calprotectin (μg/g, (**B**)), vitamin D (ng/mL, (**C**)) levels, and total LPS microbiota producers (UCF/g, (**D**)) in patients with ulcerative colitis (UC) in different age ranges. Lower zonulin (**A**) and calprotectin levels (**B**) were found in middle-aged UC group than in the youngest (n = 15). In addition, (**C**) the highest vitamin D levels were found in middle-aged group. (**D**) Higher numbers of LPS producers were observed in the oldest (n = 28) group as compared to youngest group.

The Mann-Whitney U Statistic test (MW) revealed a significant effect (T = 349,000) in terms of reducing zonulin levels among the middle-aged UC participants. In particular, the Mann-Whitney test revealed lower zonulin levels in the middle-aged group than in the youngest group (*p* < 0.05). In addition, the Mann-Whitney test revealed a tendency to decrease further in the oldest group than in the youngest (*p* = 0.076, n.s). S.E.M is the standard deviation divided by root of n (n is the size sample, see Figure 2A).

### 4.2. Calprotectin

The Mann-Whitney U Statistic showed a significant effect (T = 349,000) in terms of reducing calprotectin levels in the middle-aged group compared to the youngest group (*p* = 0.34). Figure 2B shows mean ± standard error media for this marker (S.E.M, see Figure 2B).

### 4.3. Vitamin D

Kruskal-Wallis analysis revealed a significant effect for Vitamin D levels (H = 5.9, *p* = 0.049). The Mann-Whitney U Statistic analysis showed higher endogenous vitamin D levels in the middle-aged group than in the oldest and youngest groups (*p* = 0.048). Figure 2C shows mean ± standard error media (S.E.M, see Figure 2C).

### 4.4. Total Species Carriers of LPS

The Mann-Whitney U Statistic showed a significant effect (T = 349,000) in terms of increasing LPS production in the oldest group compared to middle-aged group (*p* < 0.05). The post-hoc Mann Whitney test confirmed higher LPS production in the oldest group than in the middle-aged cohort (*p* = 0.005). In addition, there was tendency for higher levels in older participants than younger ones (*p* = 0.07, n.s, see Figure 2D).

### 4.5. R Spearman Correlations Between Inflammatory Markers (Zonulin and Calprotectin) and Years of Ulcerative Colitis Evolution

**Figure 3:**
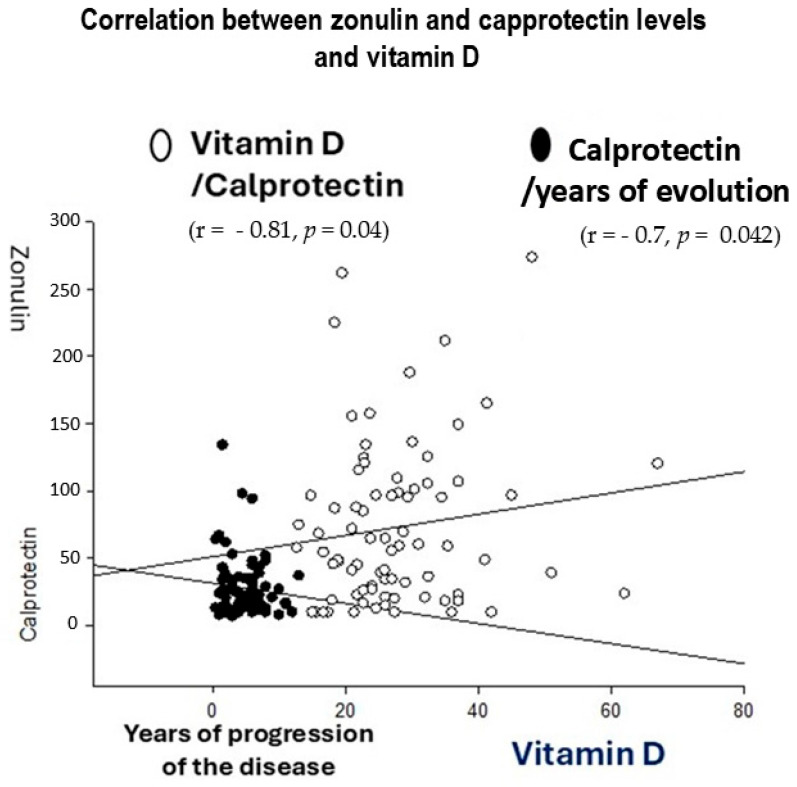
r Spearman correlations between years of ulcerative colitis (UC) progression and inflammatory markers (zonulin and calprotectin).

The r Spearman showed that endogenous vitamin D negatively correlated with zonulin levels (n = 78, r = −0.81, *p* = 0.04), which suggests protective effects against gut inflammation. In addition, calprotectin levels negatively correlated with years of UC evolution (r = −0.71, *p* = 0.042, see Figure 3).

Correlations between vitamin D and zonulin levels (left, r = −0.81, *p* = 0.04) and years of ulcerative colitis (UC) evolution and calprotectin levels were found during the r Spearman analysis (right, r = −0.71, *p* = 0.042).

### 4.6. r Spearman Correlations Between Inflammatory Markers (Zonulin and Calprotectin) and LPS Microbiota Strains Producers in Patients with Ulcerative Colitis by Age

In the middle-aged group, zonulin levels correlated with the number of LPS producers in the r Spearman analysis (r = 0.49, *p* = 0.001), which suggested the existence of strong intestinal permeability [30] or gut dysbiosis. In general, high calprotectin concentration reflects gut inflammation. In our study, *Clostridium* spp. correlated with zonulin levels in the middle-aged group ( r = 0.75, *p* = 0.00005). In this age range, the r Spearman confirmed that zonulin levels correlated with total number of microorganisms (r = 0.36, *p* = 0.037). Furthermore, there was a r Spearman correlation between vitamin D and LPS producers (r = 0,9, *p* = 0.04), which suggests that vitamin D plays a role in regulating gut immune responses [31]. In fact, zonulin and calprotectin levels correlated in middle-aged participants (r = 0.49, *p* = 0.001, see Table 1). For more correlations, see Table 1.

### 4.7. Numbers of UCF/g of Enteroccocus spp., E. Coli biovare, and Candida albicans

**Figure 4:**
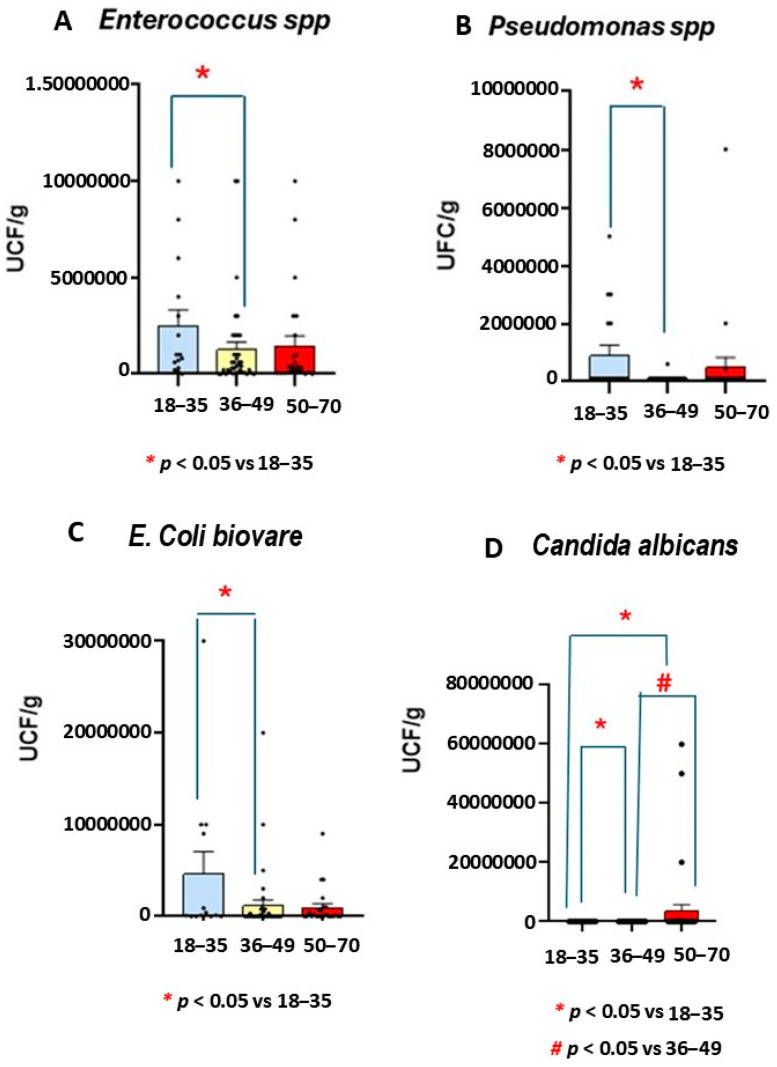
Number of *Enterococcus* spp., *Pseudomonas* spp., *E. Coli biovare*, and *Candida albicans* microbiota strains in patients with ulcerative colitis (UC) by age. The numbers of *Enterococcus* spp., *Pseudomonas* spp., *E. Coli biovare,* and *Candida albicans* were decreased in middle-aged patients compared the youngest group (n = 15, *p* < 0.05). However, increased numbers of *Candida albicans* were observed in the oldest group compared to other UC groups (*p* < 0.05 in both cases).

The Mann Whitney test revealed a lower number of *Enterococcus, E. Coli biovare*, and *Pseudomonas* spp. strains in middle-aged participants than in the youngest group (*p* = 0.049, see Figure 4).

The Kruskal-Wallis analysis confirmed not only a smaller number of *Enterococcus* spp. at middle age but also decreased number of *Pseudomonas* spp. (H = 8.37, *p* = 0.015) when compared to the youngest group (*p* = 0.004, see Figure 4).

Mann Whitney confirmed fewer *E. Coli biovare* in the middle-aged range than in the youngest participants (*p* = 0.049, see Figure 4).

However, a higher number of *Candida albicans* was found in the oldest participants than in the youngest and middle-aged groups (*p* < 0.05, Figure 4).

In addition, r Spearman analysis confirmed a positive correlation between *Candida albicans* and zonulin levels (r = 0.41, *p* = 0.047, see Table 1).

### 4.8. Analysis of Microbiota Strains by PCR

There was a tendency for the number of UCF/g for *Bifidobacterium adolescentis* to decrease in middle-aged (H = 3.89, *p* = 0.14, n.s) UC participants more than in the youngest (*p* = 0.081, n.s) and oldest (*p* = 0.074, n.s, see Table 2) groups.

The Mann-Whitney U test did not show significant effects for other microbiota strains, such as *Lactobacillus plantarum* (H = 2.56, *p* = 0.27, n.s), *Clostridium* spp. (H = 2.33, *p* = 0.312, n.s), *E. coli* (H = 0.6, *p* = 0.73, n.s), *Bacteroides* (H = 2.35; *p* = 0.3, n.s), or *Akkermansia muciniphila* (H = 1.47, *p* = 0.47; n.s, etc., see Table 2). The Mann-Whitney test revealed a tendency for the number of *Bacteroides* (UCF/g) to increase more in the oldest group compared to the middle-aged group (*p* = 0.12, n.s, see Table 2).

### 4.9. r Spearman Correlations Between LPS Producers and Rest of Markers

In the oldest participants, there were correlations between the number of LPS producers and vitamin D levels (r = −0.6, *p* = 0.0059), as well as between the number of leucocytes and vitamin D levels (r = 0.41, *p* = 0.047).

In the middle-aged participants, LPS producers correlated with *Bifidobacterium* spp. (r = 0.73, *p* = 0.00000002) and *Rummicocus* spp. (r = 0.68, *p* = 0. 029), as demonstrated by r Spearman analysis.

### 4.10. Other Parameters (PCR/Ig G/A/M)

There were no significant effects for PCR (H = 1.92; *p* = 0.38, n.s), the number of leucocytes (H = 1.48; *p* = 0.47, n.s), or Ig G, Ig M, or Ig levels among UC groups (see Table 2).

Kruskal-Wallis failed to show a significant effect for many microbiota strains (see Table 2). When parametric data were not available, we applied a Mann Whitney post-hoc test to detect significant differences. In cases of homogeneity of variance, ANOVA and Bonferroni post hoc test were applied for multiple comparisons among variables by age (*p* > 0.05 in all cases, n.s, see Table 2).

#### 4.10.1. Leucocytes (Number of Leycoytes/mm^3^)

There was no significant effect for the number of leucocytes (H = 1.48, *p* = 0.47, n.s) or PCR levels (H = 1.92; *p* = 0.38, n.s, see Table 2), as demonstrated by Kruskal-Wallis analysis.

#### 4.10.2. Statistical Analysis

Normality of data was assessed using the Shapiro–Wilk or Levene test using SPSS 13.0 software or the Sigma Plot v11.0 program. Based on these results, ANOVA (F values) was done when there was homogeneity of variance, followed by a post-hoc Bonferroni test for multiples comparisons among groups. In the case of the youngest patients, Schaffer was used for comparing groups with unequal sample sizes. 

In cases when data were not normally distributed, the Levene test showed a significant effect. Furthermore, we compared differences among UC groups through Kruskal-Wallis (H values) analysis. The Mann Whitney analyses or Dunnet’s test were applied for nonparametric variables, after appropriate correction for multiple comparisons as post hoc tests. All results are expressed as mean ± SEM (standard error media: variance/square root).

A *p*-value of less than 0.05 was considered statistically significant. The r Spearman correlation coefficient (r) assessed the correlation between two variables, including its *p* value.

## 5. Discussion

Ulcerative colitis is characterized by a chronic inflammatory state of the gastrointestinal system [1,7]. Zonulin predicts intestinal permeability, and calprotectin in feces is a reliable marker for assessing mucosal inflammation in ulcerative colitis (UC) patients [3,19]. The novelty of this study is the observation that gut inflammatory markers (zonulin and calprotectin in feces) exhibit age-dependent variation in adult patients with UC. In fact, middle-aged (36–49 years old) UC participants had higher endogenous vitamin D levels and lower zonulin and calprotectin levels than the youngest or oldest UC participants. Our findings suggest that intestinal inflammation is dependent on calprotectin levels. Thus, increased zonulin and calprotectin levels indirectly reflect enhanced trans-epithelial migration of either granulocytes, leucocytes, or monocytes in UC patients, leading to gut inflammation and dysbiosis, and vice versa [30,31]. A study with 58 pediatric patients confirmed that calprotectin level is a good marker for detecting active mucosal inflammation [32,33,34,35,36,37,38]. In a population of 602 patients with intestinal symptoms, calprotectin levels < 30 μg/g were found to be highly predictive of not having UC [39]. In fact, calprotectin is the only marker able to discriminate between mild, moderate, and severe cases of UC [32,33,34,35]. In our study, increased LPS-microbiota producers were found in the oldest (50–70) participants, which suggested strong gut inflammation in this age range. However, among middle-aged participants, high endogenous vitamin D levels could decrease gut induced-inflammation. Our hypothesis is aligned with a reliable role of serum vitamin D levels as a biomarker that is able to prevent active inflammatory in patients with irritable bowel disease (IBD) [39]. In fact, UC patients supplemented with vitamin D had lower levels of inflammatory markers of UC, such as C-reactive protein (CRP) and decreased fecal calprotectin levels [40]. Vitamin D exerts immunomodulator and anti-inflammatory effects, and a lack of it has been associated with inflammatory disease progression in IBD [30] and other immunological diseases [41]. The suggested anti-inflammatory role of vitamin D in middle-aged UC patients in our study agrees with the downregulation of certain cytokines reported in intestinal inflammation [42], which indirectly reduces gut permeability [43,44,45,46]. Nutritional vitamin D supplementation has been evaluated in several clinical studies [47,48,49,50]. Vitamin D deficiency and insufficiency (low 25-hydroxyvitamin D: 25-OH-D) have been detected in patients with UC [47]. Another study has shown that serum 25-OHD levels in umbilical cord blood are inversely correlated with fecal calprotectin concentrations in meconium [51]. This indirect evidence suggests that higher vitamin D levels appear to protect middle-aged UC patients against gut inflammation. By contrast, other studies have reported a negative correlation between vitamin D and PCR in Crohn’s disease [47,51], although PCR levels did not vary by age in our study. Although the beneficial effect of vitamin D supplementation is controversial [48,49,50], a suggested protective role of high endogenous vitamin D has been supported in rodent models of colitis [52]. Additionally, 1,25-dihydroxyvitamin D (1,25-(OH)2D induces immunomodulatory responses in the gut [53]. Since vitamin D availability regulates gut mucosal immunity [54], and lower levels of vitamin D were observed in the oldest UC participants than in the middle-aged ones in our study, we should not disregard the possibility that the oldest participants may have been more predisposed to intestinal inflammation and gut dysbiosis in our study. In this regard, vitamin D prevents the translocation of intestinal bacteria, which reduce gut dysbiosis [55,56,57,58,59]. For example, short-chain fatty acids and butyrate are beneficial metabolites for the intestine [59] which exert anti-inflammatory effects [60,61,62]. These indirect findings align with the well-known role of vitamin D as a regulator of innate and adaptive immune responses in UC patients [52,62]. In general, vitamin D deficiency has been reported in bowel diseases [63,64]. Since patients with vitamin D deficiency are more predisposed to faster progression of UC [62], we should not exclude the possibility that their high vitamin D levels may protect them against gut inflammation, given that vitamin D increases the expression of tight junction proteins and reinforces the integrity of the gastrointestinal barrier [65]. Concurring with our findings, vitamin D was used to predict the risk of developing Crohn’s disease in a cohort of 72,719 women (aged 40–73 years). Moreover, a higher 25-hydroxyvitamin D level was associated with greater remission and lower tumor necrosis factor-alpha levels (TNF alpha) in IDB patients, which could affect gut inflammatory mediators [53]. In our study, all samples for vitamin D analysis were collected in Spring, which excludesd any potential influence of seasonal variation on its levels [65]. In fact, more prevalence of UC occurs in towns with lower levels of sunlight [65]. Several studies have demonstrated that vitamin D regulates innate immune responses and contributes to the maintenance of the intestinal barrier integrity, all of which are altered in UC [30,42,66,67,68,69]. Since zonulin regulates the epithelial cell gap junctions [67,68], the high vitamin D levels observed among middle-aged UC participants in our study could have been protecting them against gut inflammation and permeabilization. This effect is also in line with enhanced colitis severity in preclinical models of UC mice suffering from dietary vitamin D restriction [70].

An altered ratio of non-pathogenic and pathogenic intestinal bacteria can contribute to reducing the absorption of certain micronutrients [71]. Another finding of our study was the reduced number of *Pseudomonas* spp., *Enterococcus* spp., and *E. Coli biovare* UCF/g found in the middle-aged group compared to other age ranges. Conversely, the oldest UC participants had the highest *Candida albicans* levels, together with increased LPS production.

The composition and quality of the resident gut microbiota contribute to maintenance and remission in UC patients [70T]. In addition, the gut microbiome regulates innate and adaptive immune system responses in humans [72,73]. Changes in certain microbiota strains are relevant at the clinical level. In our study, smaller numbers of CFC/g were found for *Enterococcus*, *E. Coli biovare*, and *Pseudomonas* strains in the middle-aged group. Some preclinical models that mimic colitis have confirmed the role of vitamin D in maintaining immune surveillance in mice [71,72,73,74], which suggests a protective role among middle-aged patients. Immune dysregulation and environmental factors could affect the number of gut microbiota strains [72] in UC [73,74]. Thus, older UC patients seem to be more susceptible to gut dysbiosis, since increased LPS production was found at this age, which increases intestinal inflammation. A study of the feces of people with UC confirmed a low number of butyrate-producing Bacteroides [75], indicating intestinal problems. Other microbiota strains, such as *Clostridium difficile*, contribute to relapse and remission in patients with Crohn’s disease and UC [76]. A meta-analysis confirmed the active role of *Clostridium*, *Faecalibacterium prausnitzii*, and *Bifidobacterium* spp. in patients with UC, which differed among those with persistent remission [77]. In addition, anti-inflammatory effects of *Faecalibacterium prausnitizii* have been found in Crohn’s disease patients [78]. Although intestinal microbiota alterations can provoke gut inflammation in rodent models of UC [79,80,81,82], a relationship between microbiota alteration and gut dysbiosis is difficult to establish in humans [76,81,83]. Finally, the competence among microorganisms for nutrients may change the intestinal pH or increase mucus production [31]. In this regard, *Candida* spp. has been identified in the intestinal mucosa of UC patients [79], which suggests that *Candida albicans* increases [83] in the older UC patients, making it age dependent. Since fungal strain-dependent inflammation has been reported in IBD [84,85], the high level of *C. albicans* observed in our study in the oldest UC participants suggests that they were the most susceptible age range. In fact, Candida more frequently colonizes the intestine of patients with a history of UC within a 5-year period [86].

## 6. Limitations and Future Perspective

The main limitation of this study was that the uneven age-group sizes (particularly of the youngest cohort) may have limited the power to draw conclusions. The possible interference of antibiotics in regulating microbiota strains at any point of the disease is another limitation. In fact, antibiotics alter the composition of bacteria in the intestinal lumen, indirectly reducing beneficial effects or hindering disease remission [80]. In addition, we could not exclude temporal effects of certain medications, surgery, or dietary factors in our study. In addition, the consumption of certain foods (with high saturated fat and/or high sugar levels) and artificial food additives can alter gut barrier permeability [77]. Finally, the potential influence of seasonal variation on vitamin D levels [65] could affect gut inflammatory levels in IBD.

The complex interplay between the gut microbiota and gastrointestinal diseases is an active research area. The short-chain fatty acids (SCFAs) produced by microbiota strengthen tight junctions in intestinal cells and may prevent gut permeability. Since IBD predisposes patients to vitamin D deficiency, the relationship between vitamin D levels and fecal microbiota species needs further investigation. Understanding the role of beneficial vs detrimental microbiota strains in gut dysbiosis could lead to better therapies against bowel diseases. However, microbiota analyses may be limited by the heterogeneity of study populations or by microbiome profiling techniques. The predictive value of new UC biomarkers, the analysis of microbiota strains, together with the use of regular screening, are strategies that would make it possible to monitor the long-term effects of therapies in BD patients [86,87,88,89,90].

## 7. Conclusions

Microbiota and gut inflammatory markers (zonulin and fecal calprotectin) exhibited age-dependent variation in patients with ulcerative colitis (UC). In fact, lower zonulin and calprotectin levels were found in middle-aged (36–49) UC patients with high endogenous vitamin D levels than in the youngest and oldest UC groups.

In addition, smaller numbers of certain microbiota strains, such as *Pseudomonas* spp., *Enterobacterium* spp., or *E Coli biovare*, were observed in the middle-aged participants than in the youngest ones. In the middle-aged group, a higher number of LPS microbiota producers were found than in the youngest and oldest groups. Finally, a higher number of *Candida albicans* and LPS were found in the oldest UC participants than in the middle-aged group.

## Figures and Tables

**Table 1:** r Spearman correlation between biomarkers in patients with ulcerative colitis by age. r Spearman correlations between vitamin D, inflammatory mediators (zonulin, calprotectin), and some microbiota strains by ages. The color gradation means the intensity of correlation (r) with its *p* value. The r value of 1 in blue means the maximal r Spearmen positive correlation between variables, while r − 1 in red indicates the maximal negative correlation.

	**LPS** **(36–49)**	**Calprotectin** **(36–49)**	***Clostridium*** **(36–49)**	** 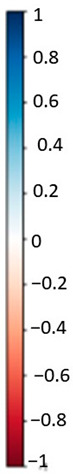 **
**Zonulin** **(36–49)**	**r = −0.69** *p* = 0.001	**r = −0.9** *p* = 0.04	**r = 0.**75 *p* = 0.00005	
	**LPS** **(50–70)**	***C. albicans*** **(50–70)**		
**Zonulin** **(50–70)**	**r = 0.41** *p* = 0.047	**r = −0.58** *p* = 0.02		
	**LPS** **(36–49)**	***E. Coli biovare*** **(36–49)**	***Clostridium* spp.** **(36–49)**	***Enterococcus* spp.** **(36–49)**
**Calprotectin** **(36–49)**	**r = 0.79** *p* = 0.0012	**r = 0.74** *p* = 0.0000001	**r = 0.75** *p* = 0.0005	**r = 0.48** *p* = 0.018
	**LPS** **(36–49)**	**Calprotectin** **(36–49)**	***E. Coli biovare*** **(36–49)**	
**Vitamin D** **(36–49)**	r = 0.68 *p* = 0.003	r = 0.5 *p* = 0.014	r = 0.74 *p* = 0.0038	
	**LPS** **(50–70)**	**Leucocytes** **(50–70)**		
**Vitamin D** **(50–70)**	**r = −0.6** *p* = 0.0059	**r = 0.41** *p* = 0.047		
	***Bifidubacterium*** **(36–49)**	***Rummicocus*** **(36–49)**		
**LPS (36–49)**	**r = 0.73** *p* = 0.00000002	**r = 0.68** *p* = 0.029		

* *p* < 0.05 indicates a statistic significant r Spearman correlation.

**Table 2:** Number of microbiota strains (CFC/g) by age. Data are expressed as a number of microbiota strains (CFC/g ± S.E.M) for other markers by age.

*Bacteroides*				
18–35		68,173,333,800 ± 6,656,031,308		H = 2.38; *p* = 0.3
36–49		1,911,106,842 ± 363,491,140		(MW: bact 36–49 vs. bact 50–70), *p* = 0.12, n.s
50–70		11,108,000,000 ± 4,633,849,803		(MW: 18–35 vs. (36–49) group, *p* = 0.1; n.s
*Lactobacillus plantarum*				
18–35		7187 ± 5812		H = 2.56, *p* = 0.27, n.s
36–49		8351.3 ± 45,412.4		
50–70		9564.8 ± 4370		
*Clostridium botulinum*				
18–35		10,000 ± 0		
36–39		33,913 ± 17,328		H = 2.33, *p* = 0.312, n.s
50–70		53,125 ± 32,583		
*E. coli*				
18–35		31,350,000 ± 15,640,119		H = 0.6, *p* = 0.73, n.s
36–39		5,633,243.2 ± 1,444,353		
50–70		7,346,538.4 ± 2,341,705.6		
*Faecalibacterium prausnitzii*				
18–35		1,961,578,947 ± 573,939,022		H = 0.224, *p* = 0.89; n.s
36–39		1,657,222,777 ± 340,905,218		
50–70		2,085,937,037 ± 537,806,296		
*E. Coli biovare*				
18–35		1,961,578,947 ± 573,939,022		H = 4.071, *p* = 0.131; n.s
36–39		1,657,222,777 ± 340,905,218 *		18–35 vs. 36–49 (*p* = 0.049, MW)
50–70		2,085,937,037 ± 537,806,296		
*Enterococcus* spp.				
18–35		2,514,000 ± 810,135		H = 3.77, *p* = 0.15; n.s
36–39		1,753,076 ± 606,510 *		
50–70		1,450,400 ± 518,619		
*Akkermansia muciniphila*				
18–35		9,182,333 ± 2,828,963		H = 1.47, *p* = 0.47; n.s
36–39		139,963,162 ± 41,909,305		
50–70		124,314,160 ± 47,206,763		
*Ruminococcus bromii*				
18–35		128,137,333 ± 66,003,115		
36–39		183,045,238 ± 43,022,266		H = 0.928, *p* = 0.62; n.s
50–70		280,800,000 ± 114,649,573		
*PCR*				
18–35		0.51 ± 102.6		H = 1.92; *p* = 0.38, n.s
36–49		0.29 ± 0.039		(MW: bact 36–49 vs. bact 50–70)
50–70		0.39 ± 0.044		
*Leucocytes* (number/mm^3^)			*pH*	
18–35		7.04 ± 0.073	6.53 + 0.22	H = 1.48; *p* = 0.47, n.s (leucocytes)
36–49		1191.63 ± 43.3	6.51 + 0.12	F (2, 64) = 0.48; *p* = 0.62; n.s (pH)
50–70		1252.61 ± 59.4	6.71 + 0.1	
	IgA	Ig G	Ig M	
18–35	104 ± 8	1120.3 ± 0.07	190.8 ± 12.49	Ig A [F (2, 64) = 0.4; *p* = 0.6; n.s]
36–49	109 ± 11	1191.6 ± 43	196.02 ± 12.8	Ig G (H = 1.48, *p* = 0.4, n.s)
50–70	111 ± 13	1252.61 ± 59	194.58 ± 10	Ig M (H = 0.218, *p* = 0.89, n.s)

**MW:** Mann-Whitney H value or ANOVA F value. **H:** Kruskall-Wallis values or Mann-Whitney values. **F:** ANOVA values. * *p* < 0.05 indicates a significant effect in comparison with youngest UC patients.

## Data Availability

All included data in this study are included within the article. Further inquiries can be directed to the corresponding authors. Data are available from the corresponding author under reasonable requirement.
